# Evaluation of machine learning models for automatic detection of DNA double strand breaks after irradiation using a γH2AX foci assay

**DOI:** 10.1371/journal.pone.0229620

**Published:** 2020-02-26

**Authors:** Tim Hohmann, Jacqueline Kessler, Dirk Vordermark, Faramarz Dehghani

**Affiliations:** 1 Institute of Anatomy and Cell Biology, Martin Luther University Halle-Wittenberg, Germany; 2 Department of Radiotherapy, Martin Luther University Halle-Wittenberg, Germany; University of South Alabama Mitchell Cancer Institute, UNITED STATES

## Abstract

Ionizing radiation induces amongst other the most critical type of DNA damage: double-strand breaks (DSBs). Efficient repair of such damage is crucial for cell survival and genomic stability. The analysis of DSB associated foci assays is often performed manually or with automatic systems. Manual evaluation is time consuming and subjective, while most automatic approaches are prone to changes in experimental conditions or to image artefacts. Here, we examined multiple machine learning models, namely a multi-layer perceptron classifier (MLP), linear support vector machine classifier (SVM), complement naive bayes classifier (cNB) and random forest classifier (RF), to correctly classify γH2AX foci in manually labeled images containing multiple types of artefacts. All models yielded reasonable agreements to the manual rating on the training images (Matthews correlation coefficient >0.4). Afterwards, the best performing models were applied on images obtained under different experimental conditions. Thereby, the MLP model produced the best results with an F1 Score >0.9. As a consequence, we have demonstrated that the used approach is sufficient to mimic manual counting and is robust against image artefacts and changes in experimental conditions.

## Introduction

The effect of irradiation on bio-chemical properties of cells or their survival can be evaluated by detection of the induced DNA damage either in the form of single strand breaks, double strand breaks or others. Double strand breaks are considered to be the most toxic form of DNA damage [1] as these lesions are significantly more complex in nature and time-consuming to repair [2,3]. A common way to visualize DNA damage or more precisely double strand breaks is via labeling of γH2AX the phosphorylated form of the H2AX histone protein variant. H2AX was shown to be part of the DNA damage signaling, located closely to DNA double-strand breaks [4,5]. γH2AX is therefore a sensitive marker for DNA damage, as the number of γH2AX foci is proportional to the dose of irradiation [6–9].

Quantifying the number of γH2AX foci is frequently conducted manually [9–12], but manual counting is laborious and the inter-rater reliability seems to be low [13–15]. Automatic and semi-automatic evaluations of γH2AX assays have the potential to overcome these issues. In the past, multiple approaches of varying complexity have been proposed [14–31]. These algorithms mostly use diverse types of image filters to highlight foci and consequently facilitate segmentation. Some also introduce object splitting to reduce effects of foci overlap [14,22,23,28,29]. The results of these approaches vary strongly in dependence on the chosen parameters and definition of a positive signal. Consequently, these algorithms often fall short reproducing manual counting for different raters. To the authors knowledge there are yet only two studies demonstrating the capabilities of machine learning to automatically adapt to manual counting for higher doses [13,31]. Herbert et al. used images with varying foci intensity and size, but used a high-quality imaging setup generating images with little noise and little to no artefacts. Furthermore, only one experimental condition has been used and changes in staining quality have not been evaluated [13]. The second study to use machine learning was designed for the analysis of low dose irradiation, discarding images of low staining quality [31]. Experimental conditions and image quality are known to impact foci density and results of automatic foci detection [9]. As many images are obtained via standard epi-fluorescence microscopy, image quality is expected to be significantly lower than in the other studies using machine learning approaches. Here, we systematically test multiple machine learning approaches, namely a multi-layer perceptron classifier (MLP), linear support vector machine classifier (SVM), complement naive bayes classifier (cNB), random forest classifier (RF) and their combinations, regarding their capability to mimic manual identification of foci areas and their count in images of low quality, containing high amounts of noise and image artefacts. We observed in our study that the MLP classifier and voting models containing the MLP classifier yield best results that can be generalized to different experimental conditions and image resolutions.

## Materials and methods

### Cell culture

For experiments the three glioma cell lines U-251 MG, LN-229 and U-343 MG were used. U-251 MG and LN-229 are glioblastoma cell lines (grade IV), while U-343 MG originates from an anaplastic astrocytoma (grade III). All cell lines were cultured in RPMI 1640 medium (Lonza, Walkersville, USA), containing 10% (v/v) fetal bovine serum (Thermo Scientific, Dreieich, DE), 1% (v/v) sodium pyruvate (Thermo Scientific, Dreieich, DE), 185 u/ml penicillin and 185 μg/ml streptomycin (Biochrome, Berlin, DE). For cell culture cells were grown at 37°C with 21% (v/v) O_2_ and 5% CO_2_ (v/v) in a humidified atmosphere.

Human glioma cells U-251MG and LN-229 both originate from grade IV glioblastoma, while U-343MG originates from grade III glioma. Cell line authentication was achieved by genetic profiling using polymorphic Short Tandem Repeat (STR) loci. Briefly, the DNA of the cell lines was isolated by GeneJET Genomic DNA Purification Kit (Thermo Scientific) according to the manufacturer's instructions. Standardized STR analyses of selected STR loci (TH01, TPOX, vWA, CSF1PO, D16S539, D7S820, D13S317 and D5S818, D3S1358, D1S1656, D6S1043, D18S51, D2S1338, D21S11, D8S1179, D12S391, D19S433, FGA, Penta D, Penta E) plus Amelogenin for gender identification of human glioma cell lines were performed at the Institute of Forensic Medicine Martin-Luther-Universität Halle Wittenberg, Laboratory of Forensic Molecular Genetics (Franzosenweg 1, 06112 Halle (Saale), Germany).

The cell cultures were tested for mycoplasma contamination at regular intervals (at least every two weeks) using the Venor^®^GeM Classic Mycoplasma PCR Detection Kit (Minerva Biolabs, Berlin, DE). For this purpose 500 μL cell culture supernatant with up to 10^6^ cells were transferred into a microcentrifuge tube, incubated for 10 min at 95°C and centrifuged for 5 s at 13.000 rpm. The resulting supernatant (2 μL) was used to prepare the PCR reaction mixture with a polymerase with a concentration of 5 U/μL according to the manufacturer's protocol.

U-251 MG and U-343 MG cells were irradiated with 2 Gy or 4 Gy 24 h before the fixation, while LN229 cells were irradiated with 2 Gy and fixed 1 h afterwards. All experiments were performed with cells in logarithmic growth phase.

### γH2AX assay

The γH2AX labelling was performed as described before [14,32]. Briefly, cells were seeded in 8-well chambers (Thermo Scientific, Dreieich, DE) for 24 h, irradiated and fixed with 4% paraformaldehyde after another 24 h or 1 h respectively. Cells were permeabilized with 0.5% Triton X-100 (Carl Roth, Karlsruhe, DE) /PBS (Lonza, Walkersville, USA) for 10 min and treated with 1% bovine serum albumin (Promega, Fitchburg, USA) for 1 h. Afterwards, the H2AX antibody (Cell Signaling, Danvers, MA, USA) and Anti-rabbit-Alexa 488 labeled secondary antibody (Alexa 488, Thermo Scientific, Dreieich, DE) were applied for 1 h. Nuclei were counterstained with DAPI (Carl Roth, Karlsruhe, DE).

Images were taken using an Axiovert 200M (Carl Zeiss, Jena, Germany) equipped with a 63x or 40x objective, resulting in images with a resolution of 1388x1038 px, corresponding to 162.6x121.6 μm or 103.2x77.2 μm, respectively.

### Detection of cell nuclei

For detection and separation of single nuclei a median filter and a contrast limited adaptive histogram equalization algorithm were applied. Afterwards, the Otsu method was used for image binarization. Overlapping nuclei were split using the watershed algorithm. The obtained nuclei were used to define regions of interest. All source codes are available as supplemental materials (S1 Source Code).

#### Detection of foci

All source codes used are available, together with a documentation file in the supplement (S1 Source Code) and on GitHub.

Model application using the final pre-trained model presented here: https://github.com/Herodot1/FociDetect

Model training: https://github.com/Herodot1/FociDetect_Training

#### Machine learning models

For foci detection 4 different models were used: a multi-layer perceptron classifier (MLP), linear support vector machine classifier (SVM), complement naive bayes classifier (cNB) and random forest classifier (RF). Furthermore, these classifiers were combined in all possible combinations using a voting classifier. To further improve the classification of each single model we used ada-boost (15 estimators) with the SVM, RF and cNB classifier. A bagging estimator (10 estimators) was used to reduce computational complexity of the SVM classification. All models and classifiers described here were obtained from the scikit-learn package v0.20.3 for python. All source codes are available as supplemental materials (S1 Source Code).

#### Generation of a feature space

As γH2AX foci differ not only from background in terms of intensity but also local gradients and potentially other properties, multiple image filters and sizes were used, mostly from the sci-kit image package v0.15 for python (exception: anisotropy filter) to generate features. The following filters were applied: Scharr filter, frangi filters (scaling range 0 to 5, stepwidth of 0.3) and gabor filters (frequencies = [0.08,0.10,0.13,0.16,0.2]). Additionally, the following filters with filter sizes of 2,3,4,5,8,10,15,20,25,30 and 35 pixels were used: auto level, auto level using the intensity percentile of 0.2 to 0.9, local histogram equalization, local minimum-maximum gradient, local minimum-maximum gradient using the percentile of 0.1 to 0.9, maximum, minimum, average, average using the percentile of 0.2 to 0.8, bilateral mean with intensity difference of ±15, median, modal, entropy and top-hat. Furthermore, an anisotropy filter with the same filter sizes as before was used [33,34].

After feature generation a principal component analysis was performed over all features and training images. Principal components cumulatively explaining 95% of the variance of the data were used to generate the final feature space, used for training the models and application of models to test-data.

#### Model training

All models were trained using 9 images of U-343 MG and U-251 MG cells irradiated with 2 or 4 Gy, and imaged using an epi-fluorescence microscope equipped with a 63x objective. The used images were the same as in a previous study of our group [14] and contained 180 cell nuclei. Each image was manually classified and pixels belonging to foci were marked for training of the models. Models were trained on 8 out of 9 images and validated on the remaining one.

For evaluation of the quality of each model we used the following metrics: F1 score and Matthews correlation coefficient [35,36]. These scores were calculated using a pixel wise evaluation. Notably, we did not use accuracy as a measure, as more than 90% of the training pixels were true negatives, making accuracy an insufficient metric.

#### Model testing

To evaluate the capabilities of the models with best scores to adapt to different conditions, we used images from LN-229 cells irradiated with 2 Gy, fixed 1 h after irradiation and imaged using an epi-fluorescence microscope equipped with a 40x objective, modulating 3 principal aspects of the experiment: spatial resolution, cell type and foci density via fixation time. Analysis of these images, containing 113 nuclei was carried out as described before, except that the images were rescaled to match the pixel size of the training images. Objects with an area smaller than 16 px were removed. For evaluation of the model quality we compared the manual foci count with the number of objects obtained for the used models. Hough-transformation was applied to adjust for object overlap in the automatic detection, to split objects and identify their centers. If the center of an automatically found focus was within the distance of 5 px (≈0.6 foci diameters) or less of the center of a manually found focus it was considered true positive, otherwise false positive. Similarly, false negative signals were detected. The false negative rate (FNR), positive predictive value (PPV), sensitivity and F1 score were calculated for model evaluation.

To verify the validity and robustness of the here used procedure we analyzed an image set containing 21 nuclei published by Herbert et al. [13]. In their publication the colleagues used budding yeast cells, labeling foci with Zip3-GFP and Msh4-GFP and images were generated using a Deltavision IX70 confocal microscope [13]. Consequently, the system is only remotely similar to the ones used so far. To allow evaluation, we first rescaled the images so that foci sizes are comparable to the ones used for training here. Afterwards, the same procedure as for the LN-229 cells irradiated with 2 Gy and fixed 1h after irradiation was performed.

### Statistics

For creating box-plots the central mark was defined as the median, the boxes showed the 25th and 75th percentile, whiskers indicated the most extreme points not considered outliers (median ± 2.7 σ, default settings in MatLab 2013a) and outliers were marked as red “+” symbols.

The 95% confidence intervals were displayed as error bars in any bar plot. Confidence intervals were constructed empirically via basic bootstrapping, regarding the detection results (true positives, false positives, false negatives) for each nucleus as an independent event. Bootstrapping was performed 1000 times per experimental group. Statistical significance was assumed, when 95% confidence intervals did not overlap. For bootstrapping and construction of confidence intervals MatLab 2013a (The MathWorks, Natick, USA) was used.

## Results

### Determination of image quality

**Fig 1:**
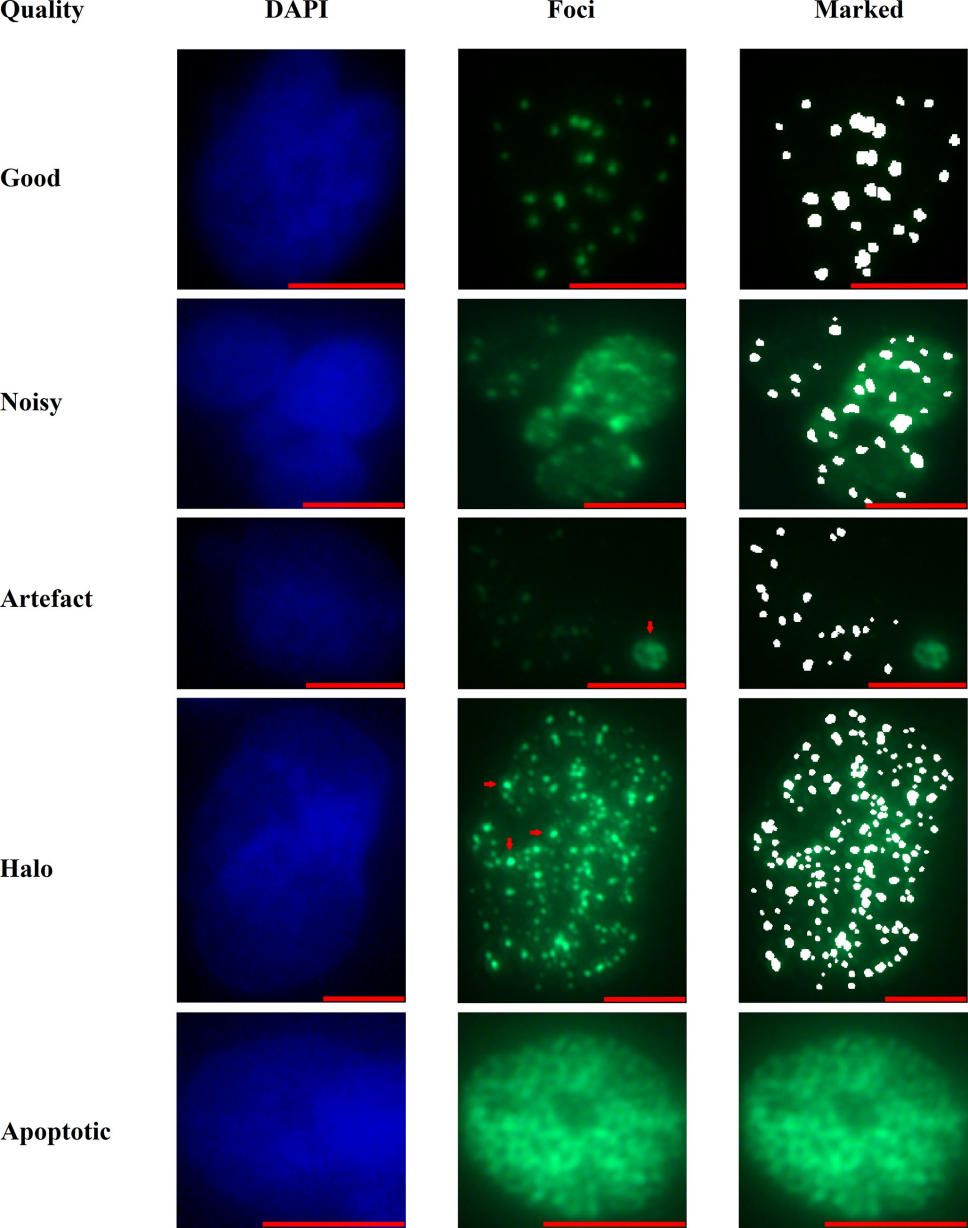
Assessment of image qualities. Diverse types of image qualities are displayed, showing the nuclear (DAPI) and γH2AX (Foci) staining, as well as the final manual markings of the respective image. Five types of anomalies are presented: high background levels or a low signal to noise ratio, labeling artefacts, halos around foci and apoptotic cells.

The quality of the used images was assessed before training the models. We defined 5 categories of image qualities: 1) good: referring to nuclei without artefacts or any disturbances 2) noisy: represents nuclei with high background or low signal to noise ratio of the foci signal, 3) artefacts: define images containing labeling artefacts, 4) halos: point to images showing halos around foci and 5) apoptotic nuclei. Examples for each type of nucleus are shown in Fig 1.

Assigning all 180 nuclei in the training images to these categories yielded that roughly 62% of all nuclei were of good quality, while 24% were considered noisy, 11% showed halos and 2% contained artefacts or were apoptotic (Table 1). Taken together, the analyzed images represent an adequate mixture of images to train the used statistical models on data obtained from subpar images.

**Table 1:** Number of nuclei and their image quality.

Quality	Good	Noisy	Artefacts	Halos	Apoptotic
Nuclei	111 (61.7 %)	44 (24.4 %)	3 (1.7 %)	19 (10.5 %)	3 (1.7 %)

### Training of statistical models

**Fig 2:**
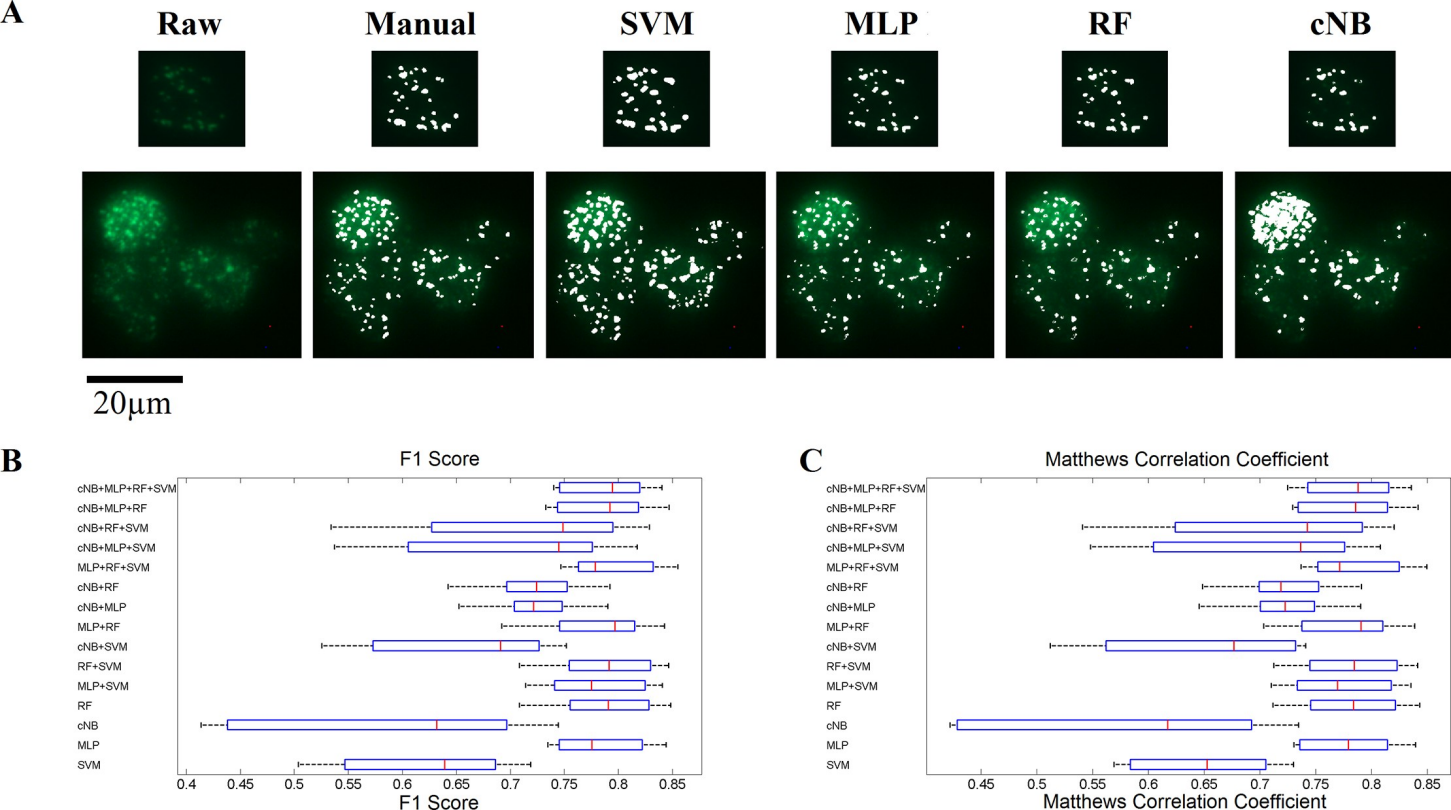
Summary of the training and validation results of all machine learning models. A) Visual comparison of different machine learning models with manual classification. All depicted models yielded reasonable results for nuclei with high signal to noise ratio, but increased noise levels lead to over fitting of the SVM and cNB model. B) and C) F1 score and Matthews correlation coefficient of all models. The SVM, cNB and most models combined with those yielded worse classification results compared to the remaining models. Box plots: the central mark corresponds to the median, the boxes show the 25th and 75th percentile, whiskery indicate the most extreme points not considered outliers.

For model training we used all filters described in the methods section together with the original image, creating a 173-dimensional feature space. To reduce the dimensionality of the feature space a principal component analysis (PCA) was performed and only the “most important” principal components explaining 95% of the total variance were employed for model training and validation. This approach reduced the dimensionality of the feature space from 173 to 15. Afterwards, all models and their combinations were trained on 8 of the 9 training images in all combinations and validated on the remaining image (Fig 2A–2C). Generally, all models performed well on nuclei with a good signal to noise ratio, without further artefacts, halos etc., as observed by visual inspection (Fig 2A, upper row). For images with lower signal to noise ratio SVM and cNB showed overfitting, while the remaining models matched manual segmentation well (Fig 2A lower row). Quantitative analysis of agreement validated the visual impression. SVM, cNB and SVM+cNB led to a reduced F1 score and MCC and the voting models cNB+SVM+RF/MLP displayed an increased spread, compared to the remaining models (Fig 2B, Table 2). Consequently, it can be concluded that cNB and SVM performed the least well, while RF and MLP performed best as single models. Likewise, voting models containing cNB and/or SVM and one other model tended to perform less well (Fig 2B). For further validation on different experimental data, we therefore used the MLP, RF, MLP+RF, MLP+RF+SVM and cNB+MLP+RF+SVM models.

**Table 2:** Pixelwise evaluation of model performance.

Model	True Negative	False Negative	True Positive	False Positive
SVM	3475866	3680	154816	172406
MLP	3621996	36440	122056	26276
cNB	3532468	46102	112394	115804
RF	3625892	37815	120681	22380
MLP+SVM	3622023	37566	120930	26249
RF+SVM	3625967	37761	120735	22305
cNB+SVM	3577436	46534	111962	70836
MLP+RF	3630833	42863	115633	17439
cNB+MLP	3633011	59455	99041	15261
cNB+RF	3634212	60311	98185	14060
MLP+RF+SVM	3620910	33093	125403	27362
cNB+MLP+SVM	3564959	26191	132305	83313
cNB+RF+SVM	3567063	24060	134436	81209
cNB+MLP+RF	3624782	38493	120003	23490
cNB+MLP+RF+SVM	3625631	38500	119996	22641

### Validation of trained models using different experimental conditions

**Fig 3:**
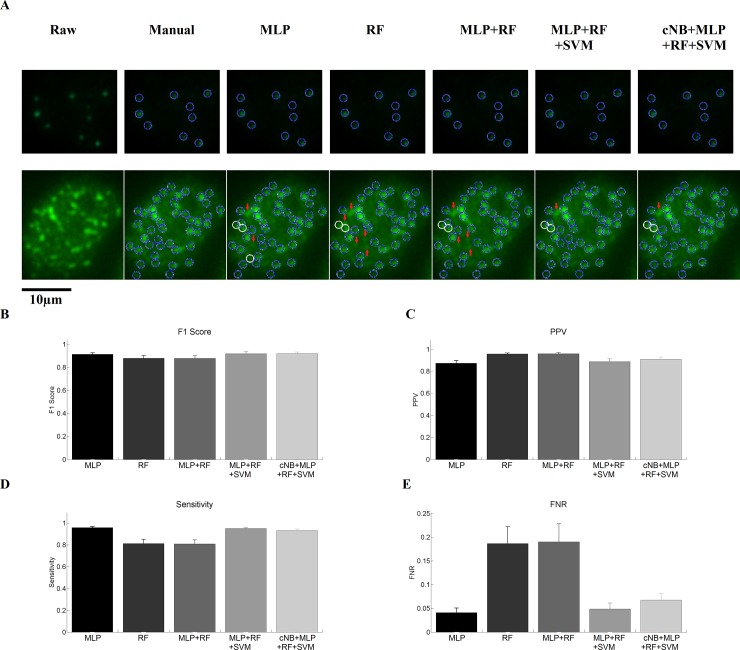
Summary of the classification results for images obtained under different experimental conditions. A) Visual comparison of different machine learning models with manual classification. All depicted models yielded reasonable results for nuclei with high signal to noise ratio, but increased noise levels lead an increase in the false negative rate of the RF and MLP+RF model. Red arrows show false negative foci, while white circles depict false positives. B)—E) F1 score, PPV, Sensitivity and FNR of all models. The RF and MLP+RF model displayed a reduced F1 score and sensitivity, but an increased PPV and FNR compared to the other models. The remaining models were indistinguishable from each other. Error bars depict 95% confidence intervals.

To evaluate whether the trained models can be extended to different experimental conditions, LN-229 cells irradiated with 2 Gy and fixed 1h after irradiation were used as they showed the highest foci number per cell creating a different foci pattern inside the nuclei. Additionally, these cells were labeled independently of the training images and were imaged with a 40x objective. These variations were done in order to demonstrate the robustness of the used machine learning models. Foci were labeled manually by the same researcher that classified the training images for comparison with the automatic results. Here, we only measured agreement in terms of identified foci. Objects identified using the automatic approach were first filtered with a size filter to remove single objects smaller than 16 px and Hough transformation was applied to identify foci centers. In a similar manner as for the training images we noticed a very good agreement of all remaining models with the manual analysis for nuclei with a good signal to noise ratio (Fig 3A, upper row), while deviations were found for subpar nuclei (Fig 3A, lower row). Quantitative analysis revealed a very good F1 score (>0.87) and sensitivity (>0.8) for all models. Notably, for the RF and MLP+RF model significantly reduced values were observed compared to the remaining models, as assessed by 95% confidence intervals (Table 3). Furthermore, the FNR (<0.20) and PPV (>0.87) of the RF and MLP+RF models were significantly increased (Table 3). This indicates that the increased PPV of the RF and RF+MLP models arises from a decreased overall number of detected foci coming at the expanse of reduced overall number of correctly identified foci (Table 4). Nevertheless, even these two models showed decent results, while the MLP, MLP+RF+SVM and cNB+MLP+RF+SVM models are virtually indistinguishable. Taken together our data indicates two main aspects: 1) the used approach can be generalized to different, but similar, experimental conditions than those used for training data. 2) The MLP, MLP+RF+SVM and cNB+MLP+RF+SVM were best suited for generalization. For further reduction of dimensionality of the feature space the MLP model was consequently used only, as this is the computationally least expensive one.

**Table 3:** Analysis of model performance.

MLP	Sensitivity	PPV	FNR	F1
Mean	0.959	0.873	0.041	0.914
95 % CI	0.947–0.969	0.848–0.896	0.031–0.053	0.900–0.928
**RF**	Sensitivity	PPV	FNR	F1
Mean	0.813	0.957	0.187	0.879
95 % CI	0.774–0.848	0.947–0.965	0.151–0.226	0.855–0.899
**MLP+RF**	Sensitivity	PPV	FNR	F1
Mean	0.810	0.959	0.190	0.878
95 % CI	0.773–0.848	0.948–0.970	0.152–0.227	0.854–0.900
**MLP+RF+ SVM**	Sensitivity	PPV	FNR	F1
Mean	0.951	0.887	0.049	0.918
95 % CI	0.938–0.964	0.861–0.910	0.036–0.061	0.901–0.932
**cNB+MLP+ RF+SVM**	Sensitivity	PPV	FNR	F1
Mean	0.932	0.907	0.068	0.920
95 % CI	0.917–0.946	0.885–0.927	0.054–0.083	0.906–0.932

**Table 4:** Object wise evaluation of model performance.

Model	False Negative	True Positive	False Positive
MLP	109	2551	372
RF	497	2163	98
MLP+RF	506	2154	91
MLP+RF+SVM	130	2530	321
cNB+MKP+RF+SVM	180	2480	253

### Reduction of parameter space

**Fig 4:**
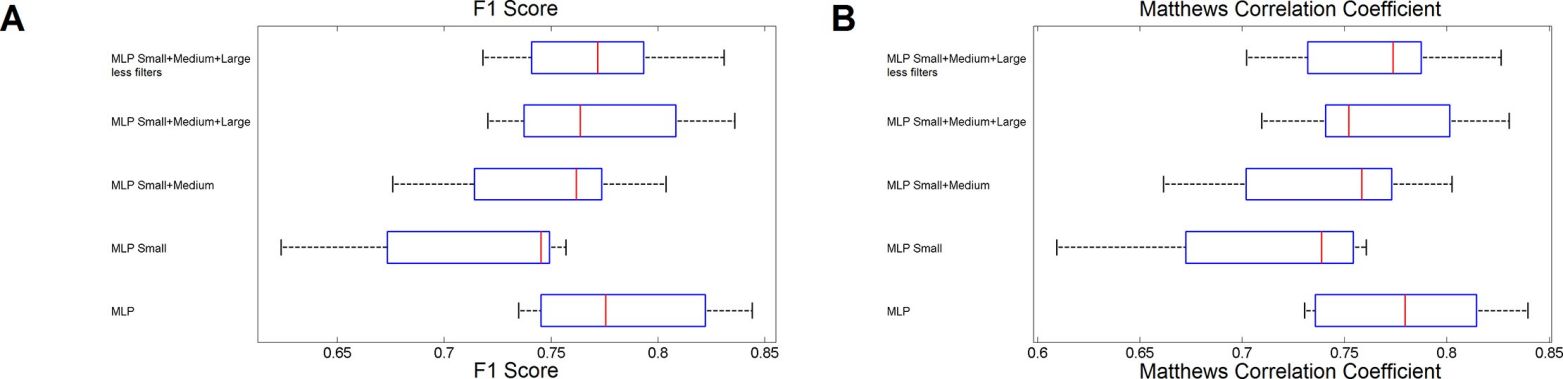
Summary of the results for the dimensionality reduction approach. A) and B) Box plots of the results for the F1 Score and MCC. Using only small or small and medium filter sizes lowers the goodness of classification. Eliminating potentially redundant filter types does not strongly impact classification results. Box plots: the central mark corresponds to the median, the boxes show the 25th and 75th percentile, whiskery indicate the most extreme points not considered outliers.

After proof of concept, the dimensionality of the input parameter space was reduced to facilitate computation. As the next step, the absolute values of the coefficients of the first two principal components were analyzed (see S1 Fig). The PCA components displayed a weak size dependent coefficient for the autolevel, autolevel percentile, tophat, entropy and anisotropy filters, indicating that results obtained by different sized filters are not entirely correlated (as expected) and compose significant information for classification. Furthermore, several different filters of the same size showed very similar coefficients, independent of their size, like autolevel and autolevel percentile filter, gradient and gradient percentile filter, mean, mean percentile, mean bilateral and median filter. These two observations indicate two possibilities: certain filters might be redundant or have very little impact and filter size is important to capture features, but potentially not all sizes. To test these hypothesizes we first reduced the number of filter sizes and frequencies to [2,3] and [0.08,0.1] or [2,10] and [0.08,0.1] or [2,10,30] and [0.08,0.16,0.2], respectively. This way, it was assured that the impact of different filter sizes was assessed. Afterwards, models were trained on the initially used training images using the reduced filter sizes. This analysis revealed that the use of small filters only results in a markedly worse classification (Fig 4). This aspect was partly compensated after the use of an additional medium sized filter (Fig 4). The application of a further large sized filter led to virtually identical classification results as the original MLP model with all filter sizes (Fig 4). Next, we removed the autolevel percentile, gradient percentile, mean percentile, mean bilateral and median filter, as these parameters provided potentially only very little additional information. Elimination of these filters did not impact the classification results significantly when compared to the original MLP filter. Consequently, we demonstrated that for classification of foci multiple filter sizes indeed lead to a gain of information, while several of the used filters do not add significant amounts of information. This approach allowed reducing the dimensionality of the initial parameter space from 173 to 36 or from 15 to 9 after PCA.

### Validation of the reduced model on an independent data set

In order to validate the applicability of the reduced model and demonstrate its robustness, we analyzed an independent earlier published image set [13], manually and afterwards with the MLP model with the reduced filter numbers and sizes. We found our approach to imitate manual counting very well, with a sensitivity of 0.9, a positive predictive value of 0.96, a false negative rate of 0.098 and a F1 score of 0.93. Empirical confidence intervals are displayed in Table 5 and a typical analysis result shown in S2 Fig. Interestingly, the F1 score obtained here is virtually identical to the best results obtained by Herbert et al. (0.925) [13].

**Table 5:** Analysis of model performance.

	Sensitivity	PPV	FNR	F1
Mean	0.902	0.958	0.098	0.923
95 % CI	0.883–0.920	0.949–0.968	0.079–0.117	0.919–0.940

## Discussion

The present study was performed to develop a method for automatic detection of γH2AX foci, using standard fluorescence microscopy, resulting in images of strongly varying quality. To assess the goodness of our approach we compared manual classification with the results of various machine learning models. Generally, a very good agreement was found between manual and automatic classification, verifying the applicability of our approach. Additionally, the used models were surprisingly robust against artefacts and changes in experimental conditions, potentially allowing a generalization of the best performing MLP model, after initial training.

### Comparison to other Detection Algorithms

Although various other automated foci counting models exist, many of them are limited in their applicability, when it comes to deal with variations in image quality or mimicking different manual raters [9,13,14]. These limitations arise in part by the way these algorithms are constructed. Many approaches use a single noise filter and a subsequent global threshold definition [16,19–21,25,26] or an additional object splitting step [22,23] to identify all remaining objects as foci. Others use local thresholds after image processing [17,24] or fully automated approaches that rely on noise filters, gradient operators, Hough transformation or wavelet transforms [14,27–29]. Most of these approaches share a certain fixed but low number of parameters that have to be manually tuned to optimize the results. This is necessary to allow an easy usage of each of these constructs. Otherwise, introducing more parameters would render parameter optimization very laborious. Nevertheless, the small parameter space potentially does not capture all information necessary to optimally fit the user idea of foci detection, as foci detection might need a higher dimensional parameter space, especially in cases of subpar image quality. This concept is in good agreement with our results, showing that different filters and filter sizes indeed capture more and diverse information, leading to a better classification. To the authors knowledge, there are two approaches that introduced machine learning for foci detection [13,31]. The study of Herbert et al. obtained training data from confocal images that did not contain any artefacts in the foci space [13]. Consequently, the images used were of much higher quality. Interestingly, the reported F1 scores were similar to the ones reported here for the same dataset, when evaluating foci numbers. This indicates that our approach is highly robust, as the used model was not trained on any of those images and the experimental settings (species, labeling, imaging device) were largely different. The study of Lengert et al. designed an algorithm for the analysis of cells exposed to very low doses of irradiation with a low foci number [31]. In their experiments cells were stained with both γH2AX and 53BP1 to label DSBs, and used both labels to generate a feature space, as true positive DSB signals had to occur in both channels. Consequently, images with noisy or weak foci staining in one channel were discarded [31]. This means that high quality images were needed, in contrast to our approach that handles different staining qualities. Nevertheless, the approach of Lengert et al. and ours cannot easily be compared, as they were designed for different purposes.

Taken together the final MLP approach, using only three filter sizes and the reduced number of filters, presented in our study can be considered to be more adaptive to experimenter specific definitions of positive signals compared to non-machine learning models. This is illustrated when comparing the results obtained here with the ones obtained in a previous study of our lab [14]. By using the same images and application of multiple foci detection approaches we demonstrated the weakness of those approaches to deal with varying image qualities [14]. Compared to other studies using machine learning we demonstrated that our final MLP model seems to be more robust to varying image qualities and experimental variations.

### Limitations

Despite the seemingly broad applicability of the presented procedure, its usage is restricted in some cases. The classification of pixels only gives the decision whether a certain pixel belongs to a focus or not. Problems associated with foci overlap due to clustering remain unsolved. In these cases additional object splitting needs to be performed. Here, we used a Hough transformation for object splitting based on the assumption of a roughly circular geometry of foci. Nevertheless, circular geometry might not be given for cells imaged shortly after irradiation. Consequently, the presented algorithm should not be applied without an adequate strategy for object splitting if high foci densities are expected. Furthermore, in our training scenarios apoptotic cells with nuclei displaying large overexposed structures in the foci channel and overexposed artefacts were sometimes misclassified. This indicates that a few bright, large structures in the foci channel that are not true positive signals might lead to misclassification of over-illuminated bright, clustered foci. Consequently, overexposure of true positive signals should be avoided during image acquisition. Notably, the approach presented here was applied to low quality images. Using images of higher quality by using confocal or super resolution imaging or discarding images of low quality will greatly enhance classification results and potentially needs a much lower dimensional feature space.

## Conclusion

In this study, the performance of multiple classical machine learning approaches was tested on a high dimensional feature space to correctly identify γH2AX foci in fluorescently labeled images. We found all models to give at least reasonable classification results and the MLP model to perform best. We thereby provided a framework for foci detection that is robust against different types of artefacts, as well as experimental changes and improves the earlier results of other automatic foci analysis software.

## Supporting information

S1 Source code Source code for model training and application and documentation. (RAR)

S1 Fig Graphical representation of the first two principal components of the initial 173-dimensional feature space. It can be seen that certain filters, such as e.g. autolevel, local histogram or the anisotropy filter show distinct size dependence. Furthermore, some filters, like autolevel and autolevel percentile filters seem to be very strongly correlated and thus potentially redundant.
(PNG)

S2 Fig Application of the reduced MLP model to external dataset. Analysis of the original images published by Herbert et al. [13]. Manual classification of foci and with the reduced MLP model revealed similar results.
(TIF)
