# Supplementary figures and images for: Evaluation of machine learning models for automatic detection of DNA double strand breaks after irradiation using a γH2AX foci assay
Source: PLoS One. 2020 Feb 26;15(2):e0229620. doi: 10.1371/journal.pone.0229620 (PMC7043763; doi:10.1371/journal.pone.0229620)

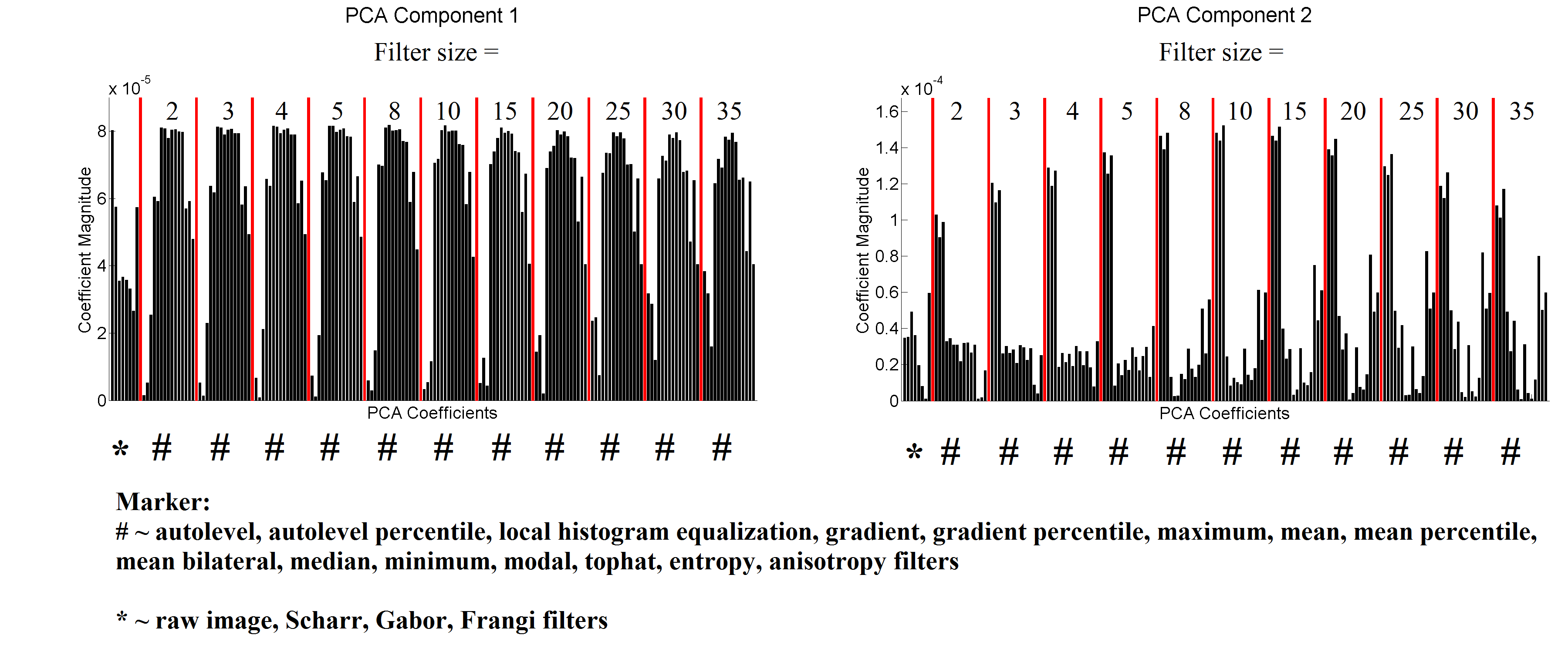

Supplement: S1 Fig — It can be seen that certain filters, such as e.g. autolevel, local histogram or the anisotropy filter show distinct size dependence. Furthermore, some filters, like autolevel and autolevel percentile filters seem to be very strongly correlated and thus potentially redundant. (PNG) [file pone.0229620.s002.png]

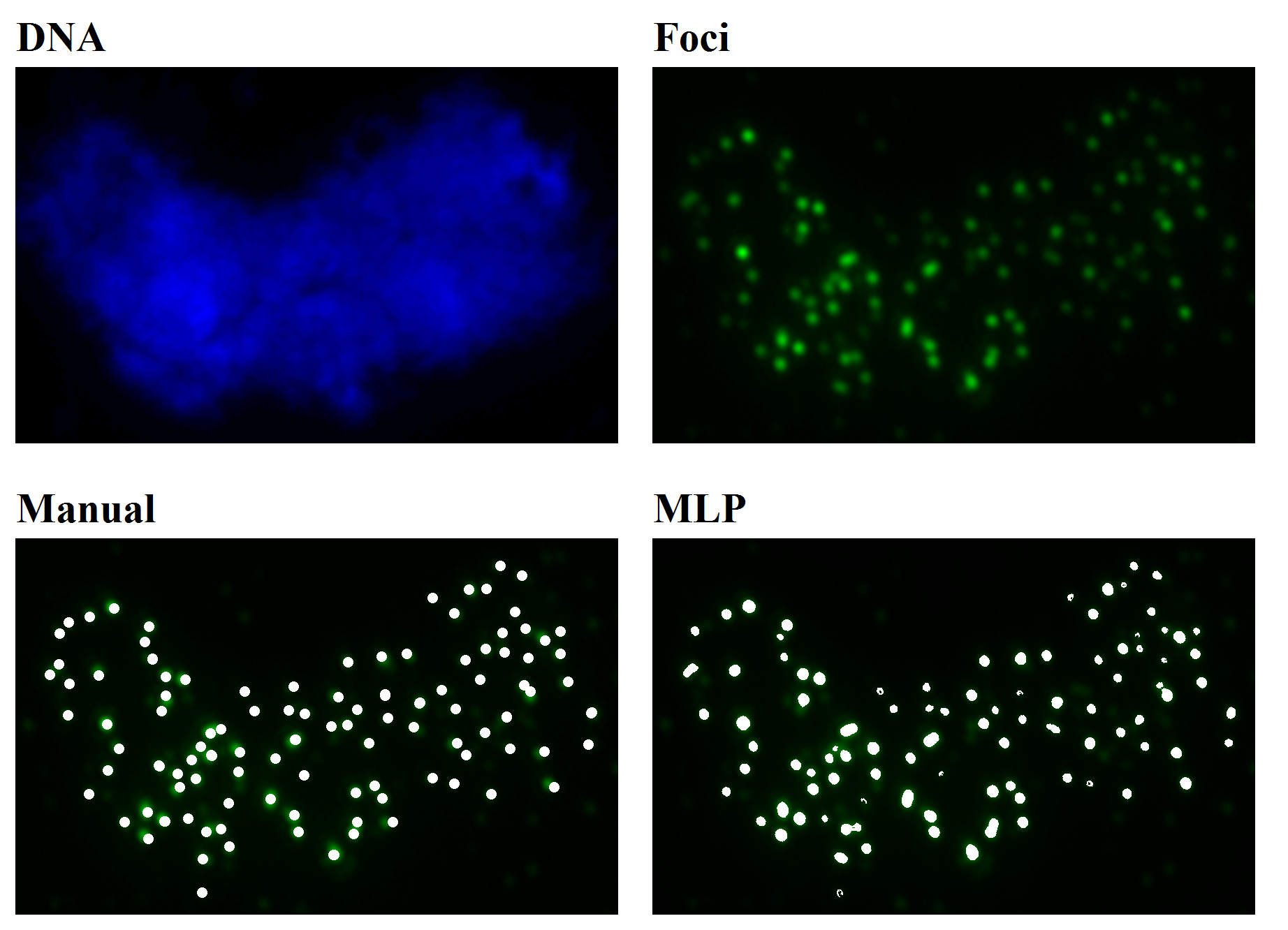

Supplement: S2 Fig — Analysis of the original images published by Herbert et al. [13]. Manual classification of foci and with the reduced MLP model revealed similar results. (TIF) [file pone.0229620.s003.tif]
